# Selective Transcription Factor Blockade Reduces Human Retinal Endothelial Cell Expression of Intercellular Adhesion Molecule-1 and Leukocyte Binding

**DOI:** 10.3390/ijms24043304

**Published:** 2023-02-07

**Authors:** Yuefang Ma, Liam M. Ashander, Binoy Appukuttan, Feargal J. Ryan, Alwin C. R. Tan, Janet M. Matthews, Michael Z. Michael, David J. Lynn, Justine R. Smith

**Affiliations:** 1 College of Medicine and Public Health, Flinders University, Bedford Park, SA 5042, Australia; 2 Precision Medicine Theme, South Australian Health & Medical Research Institute, Adelaide, SA 5000, Australia

**Keywords:** human, uveitis, retina, endothelium, leukocyte, transcription factor, C2CD4B, IRF1

## Abstract

The interaction between leukocytes and cytokine-activated retinal endothelium is an initiating step in non-infectious uveitis involving the posterior eye, mediated by cell adhesion molecules. However, because cell adhesion molecules are required for immune surveillance, therapeutic interventions would ideally be employed indirectly. Using 28 primary human retinal endothelial cell isolates, this study sought to identify transcription factor targets for reducing levels of the key retinal endothelial cell adhesion molecule, intercellular adhesion molecule (ICAM)-1, and limiting leukocyte binding to the retinal endothelium. Five candidate transcription factors—C2CD4B, EGR3, FOSB, IRF1, and JUNB—were identified by differential expression analysis of a transcriptome generated from IL-1β- or TNF-α-stimulated human retinal endothelial cells, interpreted in the context of the published literature. Further filtering involved molecular studies: of the five candidates, C2CD4B and IRF1 consistently demonstrated extended induction in IL-1β- or TNF-α-activated retinal endothelial cells and demonstrated a significant decrease in both ICAM-1 transcript and ICAM-1 membrane-bound protein expression by cytokine-activated retinal endothelial cells following treatment with small interfering RNA. RNA interference of C2CD4B or IRF1 significantly reduced leukocyte binding in a majority of human retinal endothelial cell isolates stimulated by IL-1β or TNF-α. Our observations suggest that the transcription factors C2CD4B and IRF1 may be potential drug targets for limiting leukocyte–retinal endothelial cell interactions in non-infectious uveitis involving the posterior eye.

## 1. Introduction

Non-infectious uveitis is a group of autoimmune and autoinflammatory eye diseases [1]. Although it is uncommon, non-infectious uveitis often impacts the vision, particularly when the inflammation involves the posterior segment of the eye: approximately 70% of patients with uveitis suffer vision loss to 20/60 or greater [2]. Most patients with intermediate, posterior, and pan non-infectious uveitis are initially treated with systemically administered corticosteroid and subsequently transitioned onto immunomodulatory drugs. Conventional drugs—such as anti-metabolites—plus low-dose prednisolone control the inflammation in just 24–65% of patients [3,4,5,6], and biologic drugs are increasingly being used, often targeting inflammatory cytokines such as tumor necrosis factor (TNF)-α and interleukin (IL)-1β [7].

Cell adhesion molecules are another potential biologic drug target in non-infectious uveitis involving the posterior eye. These molecules are expressed on endothelial cells that line tissue vasculature and on circulating leucocytes, and their interactions control the migration of the leukocytes across the endothelium into the tissue for homeostatic immune surveillance and during inflammation [8]. The migration process is often described as the ‘leukocyte adhesion cascade’, beginning with rolling of the leukocyte across the endothelium, followed by arrest and spreading of the leukocyte over the endothelium, and finally movement of the leukocyte through the endothelium. Endothelial selectins participate in the earliest stages, while immunoglobulin (Ig) superfamily members become the predominant players as migration proceeds. Drugs that block cell adhesion molecules have been used to treat inflammatory diseases, including non-infectious uveitis [9,10], but recognition of an association with progressive multifocal leukoencephalopathy has led to strict restrictions on their use [11]. This fatal disease is caused by reactivation of latent human polyomavirus 2 within the central nervous system (CNS); blocking leukocyte trafficking into the CNS, thus limiting immune surveillance, may break latency of the virus [12].

During inflammation, cell adhesion molecules are upregulated on the activated endothelium, promoting extravasation of large numbers of leukocytes into the tissue [8]. This suggests the opportunity for an alternative drugging approach for non-infectious uveitis: targeting the induction of endothelial cell adhesion molecules. Leukocytes enter the posterior eye across the retinal endothelium [13]. Intercellular adhesion molecule (ICAM)-1—a member of the immunoglobulin (Ig) superfamily—is expressed at relatively high levels by human retinal endothelial cells [14,15], and it is upregulated by inflammatory stimuli [13]. Expression of ICAM-1 is regulated primarily at transcription [16]. In a proof-of-concept study using a human retinal endothelial cell line, we showed that ICAM-1 levels could be controlled by manipulation at the transcriptional level [17]. In this work, we investigate the effect on ICAM-1 expression and leukocyte-endothelial interactions of targeting candidate transcription factors in multiple human retinal endothelial cell isolates.

## 2. Results

### 2.1. Candidate Transcription Factors Induced in Cytokine-Activated Human Retinal Endothelial Cells: C2CD4B, EGR3, FOSB, IRF1, and JUNB

To identify transcription factors that might promote leukocyte interactions with activated human retinal endothelial cells, we interrogated a previously published transcriptomic dataset generated from cell isolates that had been activated by brief exposure to inflammatory cytokines [18]. Differential expression analysis comparing five primary human retinal endothelial cell isolates, treated with IL-1β or TNF-α versus no cytokine for 60 min, demonstrated a 2-fold or more increase in expression of 87 genes by IL-1β and 68 genes by TNF-α at a false discovery rate (FDR) of less than 0.05 (Supplementary Table S1). Overlay of the two lists of up-regulated molecules showed 66 were common, including 12 transcription factors (Table 1). Review of the activity of these transcription factors, as reported in the published literature, identified five candidates with the potential to induce expression of human retinal endothelial cell ICAM-1, a central cell adhesion molecule coordinating leukocyte interactions in non-infectious posterior uveitis: C2CD4B, EGR3, FOSB, IRF1, and JUNB [17,19,20,21].

### 2.2. Priority Candidate Transcription Factor Targets for Drugging Leukocyte Interactions with Activated Human Retinal Endothelial Cells: C2CD4B and IRF1

Candidate transcription factors—C2CD4B, EGR3, FOSB, IRF1, and JUNB—were further evaluated for a potential role in leukocyte–retinal endothelial cell interactions in a series of molecular studies. These studies made use of 28 primary cell isolates that were sourced from eyes of 12 men and 16 women cadaveric donors aged 35 to 77 years at death (median = 61.5 years). Time from death to cell isolation ranged from 11 to 67 h (median = 31.5 h).

**Figure 1:**
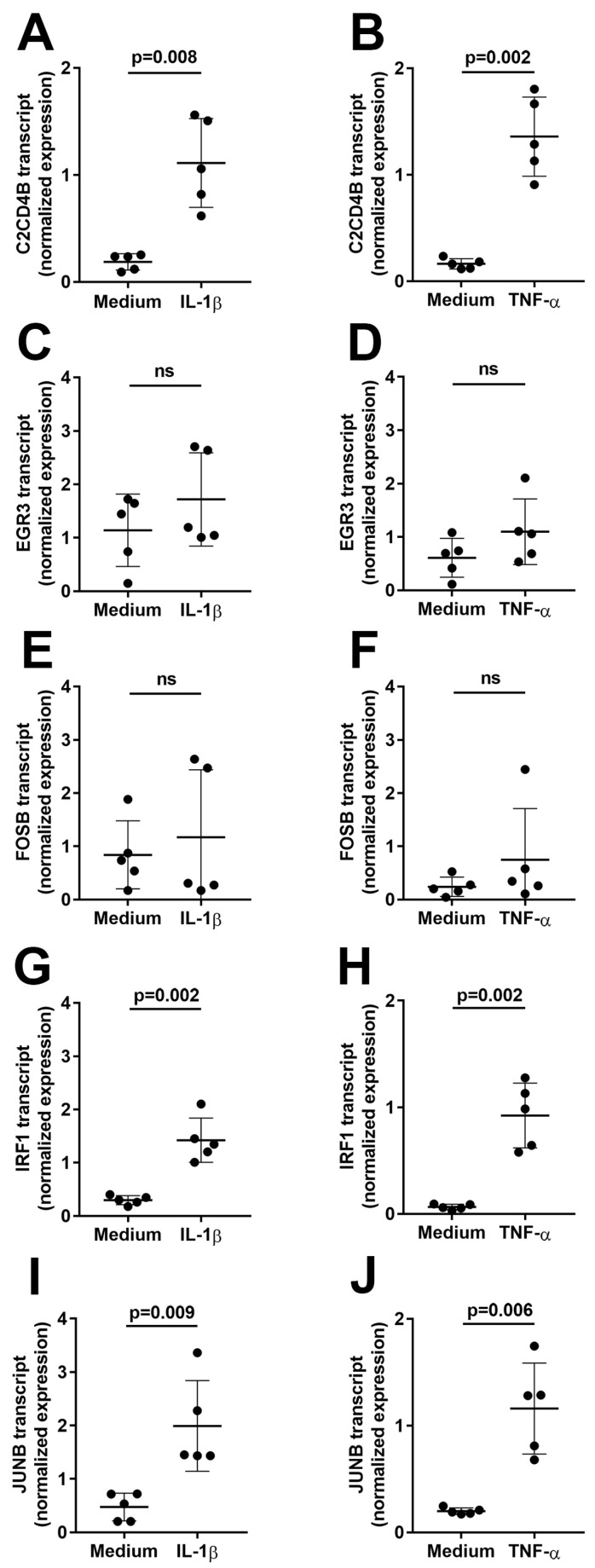
Graphs showing normalized expression of (**A**,**B**) C2CD4B, (**C**,**D**) EGR3, (**E**,**F**) FOSB, (**G**,**H**) IRF1, and (**I**,**J**) JUNB transcript, calculated relative to PPIA and RPLP0, in human retinal endothelial cells treated with (**A**,**C**,**E**,**G**,**I**) IL-1β or (**B**,**D**,**F**,**H**,**J**) TNF-α, or fresh medium alone for 4 h. Circles represent individual endothelial cell isolates. Crossbars indicate mean, and error bars indicate standard deviation (n = 5 primary endothelial cell isolates for each cytokine and control). Data were analyzed by paired Student’s *t*-test. ns = not significant.

First, to assess induction of the transcription factors in human retinal endothelial cells over an extended interval, confluent primary cell isolates were treated with IL-1β or TNF-α, or fresh medium alone as control (n = 5 isolates for each cytokine comparison) for 4 h, and gene expression levels were evaluated by reverse transcription (RT)-polymerase chain reaction (PCR). As shown in Figure 1, the expression of C2CD4B, IRF1, and JUNB was significantly increased (*p* ≤ 0.009) by treatment with IL-1β (Figure 1A,G,I) or TNF-α (Figure 1B,H,J). In contrast, there was no significant change in expression for EGR3 (Figure 1C,D) or FOSB (Figure 1E,F) in cells treated with either cytokine (*p* > 0.05).

**Figure 2:**
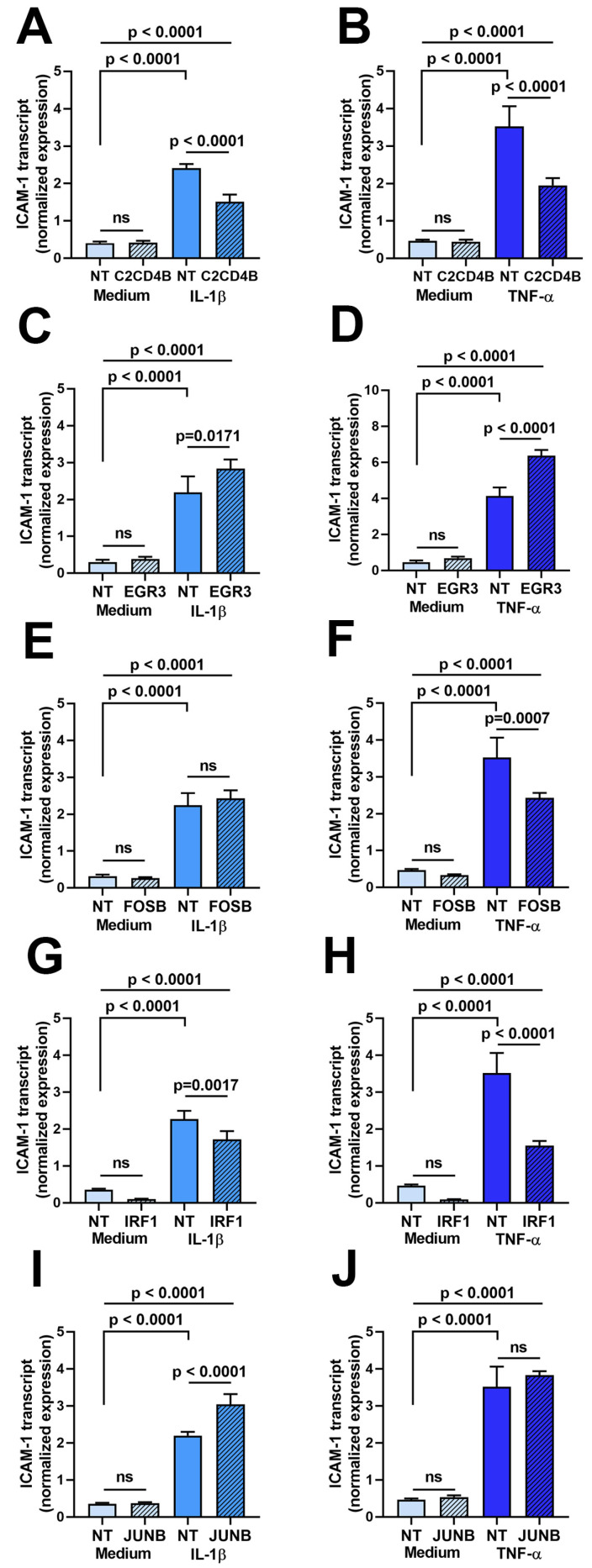
Graphs showing normalized expression of ICAM-1 transcript, calculated relative to ALAS and PPIA, in human retinal endothelial cells treated for 48 h with siRNA targeting (**A**,**B**) C2CD4B, (**C**,**D**) EGR3, (**E**,**F**) FOSB, (**G**,**H**) IRF1, and (**I**,**J**) JUNB or control non-targeted (NT) siRNA and subsequently stimulated for 24 h with (**A**,**C**,**E**,**G**,**I**) IL-1β or (**B**,**D**,**F**,**H**,**J**) TNF-α, or fresh medium alone. Bars indicate mean, and error bars indicate standard deviation (n = 4 endothelial cell monolayers per condition). Data were analyzed by one-way ANOVA with Dunnett’s multiple comparison test applied to compare individual groups.

Second, the effect of transcription factor blockade on ICAM-1 transcript expression was evaluated in a characterized human retinal endothelial cell line generated in-house. The cell line was used due to the large number of cells that were needed for the assays. As presented in Figure 2, a 24 h stimulation with either IL-1β and TNF-α, following on from a 48 h treatment with small interfering (si)RNA, increased ICAM-1 transcript expression in retinal endothelial cells. There was no significant change (*p* > 0.05) in the expression of ICAM-1 transcript in unstimulated human retinal endothelial cells following silencing of any of the five transcription factors. However, targeted siRNA knockdown of C2CD4B (Figure 2A,B) and IRF1 (Figure 2G,H) significantly decreased (*p* ≤ 0.001) the level of ICAM-1 transcript compared to non-targeted siRNA treatment under both IL-1β- and TNF-α-stimulated conditions. Knockdown of FOSB (Figure 2E,F) significantly reduced expression of ICAM-1 after TNF-α, but not IL-1β exposure, in comparison to control (*p* < 0.007 and *p* > 0.05, respectively). Expression of ICAM-1 was either unchanged (*p* > 0.05) or significantly up-regulated (*p* ≤ 0.0171) compared to non-targeted siRNA treatment when EGFR3 (Figure 2C,D) or JUNB (Figure 2I,J) were targeted under IL-1β- and TNF-α-stimulated conditions.

**Figure 3:**
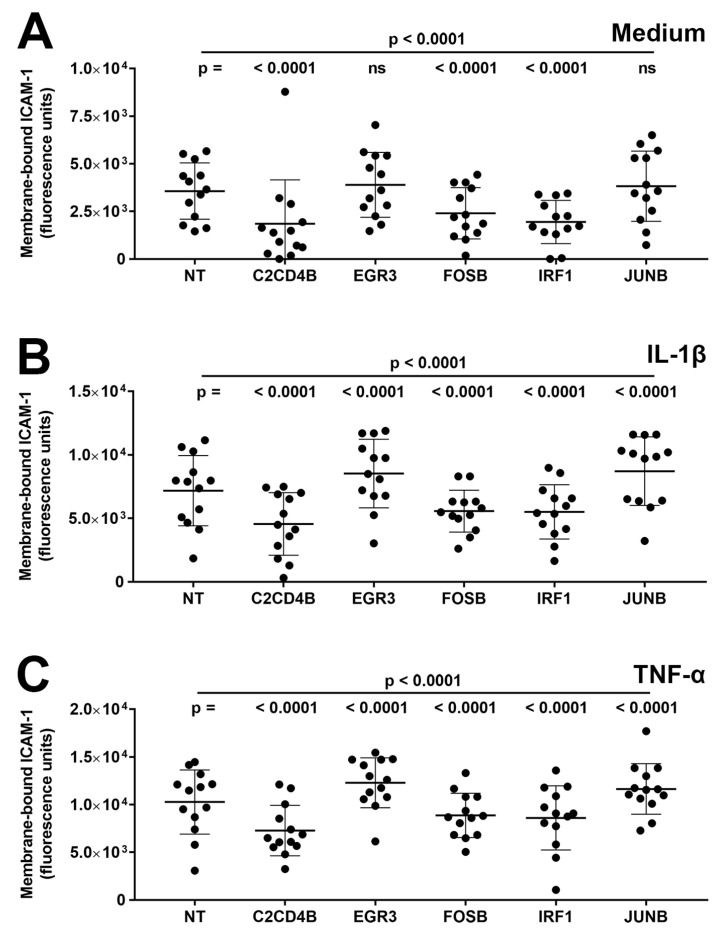
Graphs showing human retinal endothelial membrane-bound ICAM-1 protein, expressed in fluorescence units, measured after endothelial cells were treated for 48 h with siRNA targeting one transcription factor—C2CD4B, EGR3, FOSB, IRF1, and JUNB—or control non-targeted (NT) siRNA and subsequently exposed for 24 h to (**A**) fresh medium alone, (**B**) IL-1β, or (**C**) TNF-α. Circles represent individual endothelial cell isolates. Crossbars indicate mean, and error bars indicate standard deviation (n = 13 primary endothelial cell isolates under all conditions). Data were analyzed by two-way ANOVA with Tukey’s multiple comparison test applied to compare individual groups. ns = not significant.

Third, the effect of transcription factor blockade on expression levels of ICAM-1 protein on the cell membrane, representing adhesion molecule available for leukocyte binding, was measured in primary human retinal endothelial cell isolates. Cells were treated with siRNA for 48 h and subsequently stimulated for 24 h with either IL-1β or TNF-α, or fresh medium only. At the end of the assay, membrane-bound ICAM-1 was labeled by indirect immunofluorescence and measured with adjustment for cell number. Membrane-bound ICAM-1 expression was induced under IL-1β- and TNF-α-stimulated conditions, compared with the medium control, and as illustrated in Figure 3, silencing of C2CD4B, FOSB, and IRF1 significantly decreased (*p* ≤ 0.001) the level of membrane-bound ICAM-1 compared to non-targeted siRNA under all conditions (Figure 3A–C). For reduced EGFR3 and JUNB, expression of membrane-bound ICAM-1 was unchanged (*p* > 0.05) under the control condition (Figure 3A) and significantly up-regulated (*p* < 0.0001) under IL-1β- and TNF-α-stimulated conditions (Figure 3B,C).

In summary, C2CD4B and IRF1 consistently demonstrated: (1) extended cytokine-induced gene expression in activated human retinal endothelial cells; (2) decrease in cellular ICAM-1 transcript expression when silenced under cytokine-activated conditions; and (3) decrease in membrane-bound ICAM-1 protein when targeted. Thus, these two transcription factors were priority candidates for evaluating the impact of transcription factor blockade on leukocyte–retinal endothelial cell interactions.

### 2.3. Effect of C2CD4B and IRF1 Targeting on Leukocyte Interactions with Human Retinal Endothelial Cells

**Figure 4:**
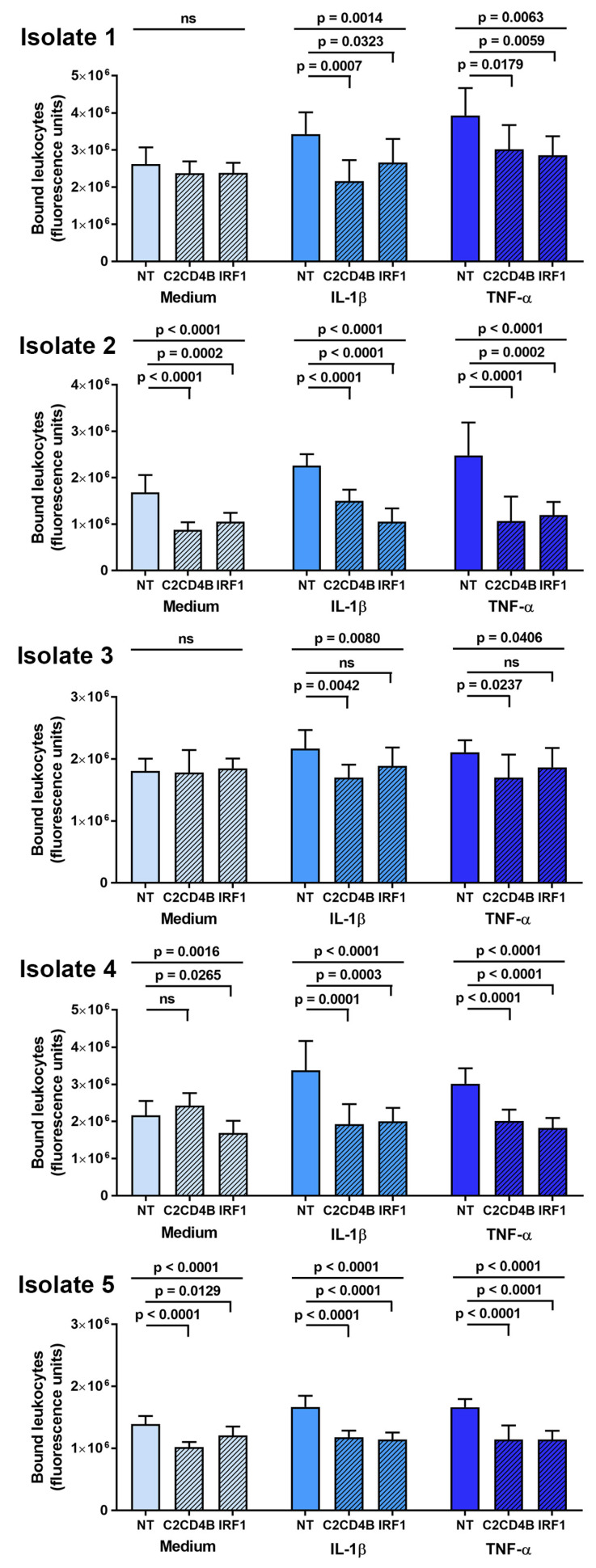
Graphs showing leukocyte binding to human retinal endothelial cell monolayers, expressed in fluorescence units, measured after endothelial cells were treated for 48 h with siRNA targeting one transcription factor—C2CD4B or IRF1—or control non-targeted (NT) siRNA, and subsequently exposed for 24 h to fresh medium alone, IL-1β, or TNF-α. Results for each primary endothelial cell isolate are presented separately. Bars indicate mean, and error bars indicate standard deviation (n = 7–8 endothelial cell monolayers per condition). Data were analyzed by one-way ANOVA with Dunnett’s multiple comparison test applied to compare individual groups. ns = not significant.

The effect of C2CD4B and IRF1 blockade on leukocyte interactions with human retinal endothelial cells was studied in a binding assay conducted under simulated flow conditions. After a 48 h treatment with targeted or control non-targeted siRNA, and a 24 h stimulation with IL-1β or TNF-α, or medium alone, individual human retinal endothelial cell isolates were rotated briefly with CFSE-labeled THP-1 leukocytes. Leukocyte binding was measured as fluorescence of the co-cultures. As shown in Figure 4, targeted knockdown of C2CD4B led to significant reduction in THP-1 leukocyte binding across five individual human retinal endothelial cell isolates stimulated by IL-1β or TNF-α, in comparison to the non-targeted siRNA control treatment. For IRF1, the same result was observed in four of five isolates. In assays performed with non-activated endothelial cells treated with a fresh medium without cytokine, there was no significant change (*p* > 0.05) in THP-1 leukocyte binding for three of five isolates when C2CD4B was targeted and for two of five isolates when IRF1 was targeted. These results suggest that blockade of C2CD4B or IRF1 in cytokine-activated human retinal endothelial cells inhibits leukocyte binding for a majority of donors.

## 3. Discussion

The central role of transcription factors in cell identity and activity, and in the development and progression of diverse diseases including uveitis [22], led to early enthusiasm around therapeutic targeting of gene transcription [23]. However, features of transcription factor interactions with DNA and co-factors—elements of disorder or ‘fuzziness’ without structured binding pockets [24,25]—limited progress in the field for decades, and there was a general consensus that transcription was undruggable. Recent developments that promise to reverse this perspective include new knowledge of intrinsically disordered proteins, cellular thermal shift assays to interrogate target engagement, binding-focused drug screening methodologies, proteolysis targeting chimera-based therapeutics, and CRISPR-directed transcriptional interventions [26,27]. In the area of medical oncology in particular, numerous therapeutics that target transcription factor activity are currently under development or have already entered clinical trials [28]. In this work, we show that targeting the transcription factors, C2CD4B, and IRF1, limits interactions between human retinal endothelial cells and leukocytes, which is a key initiating step in non-infectious uveitis involving the posterior eye.

At the time of writing, C2CD4B remains quite poorly characterized among transcription factors, with less than 30 peer-reviewed articles published on this molecule, and no articles reporting its activity in retinal endothelial cells from humans or any other mammalian species. Originally described in 2004 and named nuclear localized factor (NFL)2, C2CD4B was measured at relatively high levels in human endothelial cells in comparison to epithelial cells, smooth muscle cells, fibroblasts, and monocytes, and was found to be induced by IL-1β and TNF-α [19]. Subsequent genetics-focused research has linked C2CD4B to glucose homeostasis, with increased activity promoting the development of type 2 diabetes mellitus by mechanisms that may be sexually dimorphic [29,30,31], and to regulation of coagulation factor VII and von Willebrand factor, with a potential causal role in vascular occlusive disease [32]. Interestingly, although most studied in relation to non-infectious uveitis, interactions between leukocytes and the retinal endothelium also occur in diabetic retinopathy and retinal vein occlusion [33,34], suggesting a role for C2CD4B beyond immune-mediated eye disease.

In contrast to C2CD4B, IRF1 has been extensively researched, regulating expression of multiple genes involved in adaptive and/or innate immunity, as well as inflammatory diseases [35]. Polymorphisms in the *IRF1* gene have been associated with Behçet disease, which often presents with uveitis [36]. Consistent with our results, Guo and colleagues showed parallel up-regulation of both IRF1 and ICAM-1 in rat retinal endothelial cells activated by mitochondrial DNA, which is a damage-associated molecular pattern (DAMP) [37]. Although they did not examine the regulatory relationship or leukocyte binding, there is evidence of IRF1 promoting ICAM-1-mediated leukocyte-endothelial cell interactions in other experimental systems, for example, cerebral malaria in *Irf1* gene-deficient mice [38]. However, there is also evidence of vascular endothelial cell heterogeneity in relation to IRF1-mediated effects on interactions with leukocytes: in a study involving human umbilical vein endothelial cells, Yan and co-workers found that IRF1 silencing reduced the expression of vascular cell adhesion molecule (VCAM)-1 but did not alter the expression of ICAM-1 [39].

Unexpectedly, ICAM-1 was induced in cytokine-activated human retinal endothelial cells after knockdown of EGR3 and JUNB. We speculate that loss of the effect of these transcription factors may trigger a feedback mechanism, whereby other activators over-compensate for the change. It is also possible that EGFR3 and JUNB have human retinal endothelial cell-specific repressive activity. Further molecular analyses would be required to characterize this phenomenon. Since our interest was the blockade of the interaction between leukocytes and retinal endothelium, these transcription factors were not studied in the leukocyte binding assay.

A major strength of this work is the use of multiple human retinal endothelial cell isolates. The mouse is commonly employed in research on retinal diseases. Detailed comparisons of the transcriptomes of mouse and human tissues have revealed considerable differences between the two species, leading to the conclusion that “regulatory information in general, such as transcription factor binding, is highly diverged” [40]. Thus, for valid translation, it was important we employed a human system, although this necessarily required in vitro assays. In general, published research with human retinal endothelial cells uses a single isolate [18]. However, interindividual variation is an important consideration: for one of five human retinal endothelial cell isolates we studied, IRF1 silencing did not significantly reduce leukocyte binding, even while C2CD4B silencing did. Across this work, we used 28 human retinal endothelial cell isolates generated from women and men donors, to ensure consistent results.

Our approach to identifying priority target transcription factors involved an initial in silico analysis, follow-up molecular studies focused on ICAM-1 expression, and final cellular assays. Thus, from numerous potential targets, we identified two key transcription factors that clearly impacted leukocyte binding to activated human retinal endothelial cells. Notably, for three or two of these five isolates, respectively, targeting C2CD4B or IRF1 did not impact binding under non-activated basal conditions. This observation implies that it may be possible to limit leukocyte binding in the diseased condition primarily, without impacting homeostatic leukocyte trafficking and immune surveillance. Consistently, C2CD4B and IRF1 silencing had limited or no impact on baseline retinal endothelial cell ICAM-1 expression in the molecular studies. There are several ICAM-1 targeted therapeutics that have either been trialed in humans (e.g., alicaforsen, an ICAM-1 anti-sense oligonucleotide [41], and BI-505, an anti-ICAM-1 monoclonal antibody [42]) or are being evaluated in pre-clinical studies (e.g., ICAM-1-specific chimeric antigen receptor T cell therapy [43] and 3DNA nanocarrier-conjugated anti-ICAM-1 antibody [44]); however, these differ from the transcription factor-directed drugging strategy proposed here.

The goal of our research was to identify transcription factors that impacted leukocyte binding to human retinal endothelium and therefore might represent attractive drug targets for non-infectious uveitis involving the posterior segment of the eye. Members of the Ig superfamily coordinate leukocyte firm adhesion to endothelium, as well as initiation of leukocyte diapedesis [45]. Previous research has identified ICAM-1 as the dominant Ig superfamily member for migration of lymphocyte subsets involved in non-infectious uveitis across human retinal endothelium: ICAM-1 blocking antibody reduces human retinal endothelial transmigration of Th1 cells, Th17 cells, and B cells in most humans [46,47]. We have also observed a key role for ICAM-1 in human retinal endothelial cell-monocyte interactions (unpublished data). Therefore, our molecular studies focused on ICAM-1. It is possible that C2CD4B and/or IRF-1 also influence the expression of other retinal endothelial cell adhesion molecules. The Ig superfamily members, VCAM-1 and activated leukocyte cell adhesion molecule (ALCAM), are expressed by human retinal endothelial cells and may be induced by inflammatory cytokines [13,48]. However, transendothelial migration studies show no role for VCAM-1 and ALCAM in lymphocyte migration into the retina in most individuals [46,47].

In summary, we have demonstrated that silencing C2CD4B or IRF1 reduces ICAM-1 expression by cytokine-activated human retinal endothelial cells and decreases leukocyte binding to these endothelial cells. Our observations suggest the possibility of developing therapeutics directed against C2CD4B or IRF1 for non-infectious uveitis involving the posterior eye. Drug formulation remains a challenge, but the eye lends itself to local drug delivery due to physical accessibility and compartmentalization, and there are numerous innovations beyond simple intraocular drug injection [46]. While non-infectious uveitis is our primary interest, the cellular interaction that we have studied occurs in several other retinal diseases—diabetic retinopathy, retinal vein occlusion, and ocular toxoplasmosis [32,33,45]—suggesting broader potential application of our findings.

## 4. Materials and Methods

### 4.1. Selection of Transcription Factor Candidates

An RNA-sequencing dataset that represented the transcriptome of human retinal endothelial cells in resting state, and TNF-α- and IL-1β-activated states (Gene Expression Omnibus, National Center for Biotechnology Information Series link: GSE144785) [18] was the source of transcription factor candidates. Using the normalized read counts, adjusted for donor effect as presented in the description of this dataset, differential gene expression analysis was performed in the EdgeR v3.26.5 package [49]. Differentially expressed genes were defined by a fold-change of at least 2, plus an FDR of less than 0.05 [50]. Cross-referencing these lists identified all transcripts that were up-regulated by both TNF-α and IL-1β at 60 min. The National Library of Medicine (NLM) National Center for Biotechnology Information (NCBI) Gene database [51] and the Johns Hopkins University Online Mendelian Inheritance in Man (OMIM) catalog [52] were used to interrogate this transcript subset to identify those that encoded transcription factors. The NLM NCBI Pubmed bibliographic search engine [51] was then used to locate peer-reviewed published evidence of an association between any of these transcription factors and the induction of ICAM-1 during inflammation. This search used the transcription factor names and ICAM-1 as search terms.

### 4.2. Human Retinal Endothelial Cells

Human retinal endothelial cell isolates were prepared from paired posterior eyecups obtained from the Eye Bank of South Australia (Adelaide, Australia), after removal of the cornea for use in transplantation. None of the donors had a history of uveitis or any other retinal disease. The isolation method, as well as phenotypic descriptions of these cells, has been detailed in previous publications [13,14,15,53]. In brief, the retina was dissected free from the posterior eyecup and digested with collagenase II in varying concentrations (Thermo Fisher Scientific-Gibco, Grand Island, NY, USA). Endothelial cells were purified from the digested tissue by magnetic selection, using Dynabeads M-450 Epoxy magnetic beads (Thermo Fisher Scientific-DYNAL, Oslo, Norway) conjugated to anti-human CD31 antibody (BD Biosciences-Pharmingen, San Diego, CA, USA). The retinal endothelial cells used in the gene expression studies were previously transduced with the LXSN16E6E7 retrovirus (gifted by Denise A. Galloway, Fred Hutchinson Cancer Institute, Seattle, WA, USA) [54], which provided the necessary numbers of cells for those studies; these cells retain an endothelial cell phenotype [13]. Unless otherwise stated, the endothelial cells were cultured in a modified MCDB-131 medium (Merck-Sigma Aldrich, St. Louis, MO, USA) that had been supplemented with 10% fetal bovine serum (FBS) (Thermo Fisher Scientific-Gibco or GE Healthcare-Hyclone, Logan, UT, USA) and endothelial growth factors (EGM-2 SingleQuots supplement, omitting FBS, hydrocortisone and gentamicin; Clonetics, Lonza, Walkersville, MD, USA) at 37 °C and 5% CO_2_ in air.

### 4.3. Small Interfering RNA, Recombinant Cytokines and Monoclonal Antibodies

The siRNAs were selected from the Silencer Select range produced by Thermo Fisher Scientific-Ambion (Austin, TX, USA): C2CD4B (siRNA ID = s229328), EGR3 (siRNA ID = s4545), FOSB (siRNA ID = s5343), IRF1 (siRNA ID = s7501), JUNB (siRNA ID = s7661), NFKB1 (siRNA ID = s9504), and Negative Control No. 1 siRNA. The GeneID [55] and sequences of the targeted siRNA are presented in Table 2. Human recombinant TNF-α and IL-1β were purchased from R&D Systems (Minneapolis, MN, USA), and used in experiments at a concentration of 5 or 10 ng/mL. Mouse monoclonal anti-human ICAM-1 antibody (clone LB-2) and mouse IgG2b_κ_ isotype negative control primary antibody (clone 27–35) were purchased from BD Biosciences-BD Pharmingen (San Jose, CA, USA), and used at a working concentration of 1 μg/mL.

### 4.4. RNA Silencing

Human retinal endothelial cells were plated for confluence in 12-well (growth area = 3.8 cm^2^) or 96-well (growth area = 0.32 cm^2^) multi-well plates in modified MCDB-131 medium, and incubated for 24 h at 37 °C and 5% CO_2_ in air. The medium was refreshed, and cells were treated with targeted or control siRNA. For each well of the 12-well plates, 1 mL of fresh medium was combined with a transfection mixture, produced by combining 12 pmol of siRNA diluted in 100 μL of Opti-MEM I Reduced Serum Medium (Thermo Fisher Scientific-Gibco) with 100 μL of Opti-MEM I containing 2 μL of Lipofectamine RNAiMAX Transfection Reagent (Thermo Fisher Scientific-Invitrogen, Carlsbad, CA, USA). For each well of the 96-well plates, 90 μL of fresh medium was combined with a transfection mixture, produced by combining 1 pmol of of siRNA diluted in 5 μL of Opti-MEM I Reduced Serum Medium with 5 μL of Opti-MEM I containing 0.3 μL of Lipofectamine RNAiMAX Transfection Reagent. Cell monolayers were incubated for 48 h at 37 °C and 5% CO_2_ in air, with a change of medium after 24 h, and subsequently treated for 24 h with an MCDB-131 medium with 10% FBS, endothelial growth factors, and either TNF-α or IL-1β, or fresh medium without cytokine as control. Effect of the Silencer Select siRNA on expression of transcription factors was confirmed by RT-PCR (Figure S1). At the end of the treatment period, the medium was removed from cell monolayers in the 12-well plates, and RLT lysis buffer (Qiagen, Hilden, Germany) containing 2-mercaptoethanol was added to each well. The plates were frozen immediately at −80 °C in preparation for RNA extraction. Cell monolayers in the 96-well plates were either used immediately for leukocyte binding assays or washed twice in phosphate-buffered saline with divalent cations (PBS), fixed in 1% paraformaldehyde for 30 min at room temperature, washed again with PBS, and stored under PBS at 4 °C for the membrane ICAM-1 immunoassay. We performed the RNA silencing step prior to the cytokine treatment to provide the cleanest assessment of transcription factor targeting, since we sought to test a therapeutic approach and not to develop a therapeutic agent. This also avoided unnecessary additional manipulations, such as the need to replenish the cytokine multiple times over the course of an experiment.

### 4.5. RNA Extraction and Reverse Transcription

RNA was extracted from the human retinal endothelial cell monolayers using the RNeasy Mini Kit (Qiagen) with optional on-column DNase I digest or TRIzol Reagent (Thermo Fisher Scientific-Ambion) and stored at −80 °C. Nucleic acid concentrations were determined by spectrophotometry on a Nanodrop 2000 (Thermo Fisher Scientific, Wilmington, DE, USA). The cDNA synthesis was performed using the iScript Reverse Transcription Supermix for RT-qPCR (Bio-Rad Laboratories, Hercules, CA, USA), with 100 ng minimum RNA input per reaction. Duplicate reactions were prepared for each sample, and the resulting cDNA was pooled for use in the PCR.

### 4.6. Real-Time Polymerase Chain Reaction

Relative quantitation real-time PCR was performed on a CFX Connect Real-Time PCR System (Bio-Rad Laboratories). In addition to SsoAdvanced Universal SYBR Green Supermix (Bio-Rad Laboratories) and nuclease-free water, each reaction contained 750 nM of the forward and the reverse primer and 2 µL of cDNA, used at up to 1:10 dilution. Cycling conditions included a pre-amplification hold of 95 °C for 30 s, followed by 40 cycles of denaturation at 95 °C for 30 s, annealing at 60 °C for 30 s, and extension and fluorescence reading at 72 °C for 30 s. Melting curves from 70 °C to 95 °C were performed for each run to confirm a single peak was produced for all primer sets, and amplicon sizes were confirmed by agarose gel electrophoresis. Relative expression of the transcripts of interest, normalized to stable reference genes (coefficient of variation less than 0.25)—5′-aminolevulinate synthase 1 (ALAS1), peptidylprolyl isomerase A (PPIA), or ribosomal protein lateral stalk subunit 0 (RPLP0)—was determined by the Pfaffl method [56]. Primer efficiency was greater than 85% for all primer sets, as determined by CFX Manager software v3.0 (Bio-Rad Laboratories) using standard curves generated by serial dilution of purified PCR products. Primer sequences and expected product sizes are shown in Table 3.

### 4.7. Membrane-Bound Intercellular Adhesion Molecule-1 Immunoassay

Fixed human retinal endothelial cell monolayers were washed twice in PBS with 0.1% Tween-20 (PBS-T) for 5 min each. The cell monolayers were blocked in 5% skim milk (Coles, Hawthorn East, Australia) in PBS for 30 min, and incubated with mouse anti-human ICAM-1 or negative control primary antibody in blocking solution for 45 min. After 3 washes in PBS-T, the cell monolayers were incubated with goat anti-mouse IgG (H + L) Alexa Fluor 488-conjugated secondary antibody (Thermo Fisher Scientific-Molecular Probes, Eugene, OR, USA) diluted to 2.5 μg/mL in blocking solution for 30 min, washed 3 times in PBS-T, and counterstained with 300 nM 4′6-diamidino-2-phenylindole-dihydrochloride (Merck-Sigma Aldrich) in PBS for 5 min. All incubations and washes were conducted at room temperature on an OM5 orbital shaker (Ratek, Boronia, Australia; 60 rpm setting). Membrane labeling and nuclear staining were measured on the VICTOR X3 microplate reader (PerkinElmer, Waltham, MA, USA) using 485 nm excitation/535 emission and 355 nm excitation/460 emission filter sets, respectively. Membrane-bound ICAM-1 protein expression was expressed as fluorescence units, adjusted for background immunolabeling detected in wells incubated with negative control primary antibody and corrected for cell number based on the measurement of nuclear staining.

### 4.8. Leukocyte Binding Assay

THP-1 leukocytes (American Type Culture Collection, Manassas, VA, USA) were labeled with 5 µM carboxyfluorescein succinimidyl ester (CFSE, Thermo Fisher Scientific-Invitrogen) in an RPMI-1640 medium (Thermo Fisher Scientific-Gibco), supplemented with 10% FBS and 0.05 mM 2-mercaptoethanol, in complete darkness at 37 °C and 5% CO_2_ in air for 20 min, followed by an additional 5 min after 1:5 dilution in PBS. The labeled THP-1 leukocytes were resuspended in a modified MCDB-131 medium, held for a minimum of 10 min, and subsequently added to human retinal endothelial cell monolayers in 96-well multi-well plates at 10^5^ cells (100 µL) per well. The plates were rotated for 30 min at room temperature on an OM5 orbital shaker (60 rpm setting). After careful removal of any non-bound THP-1 leukocytes, cell monolayers were washed gently 4 times with PBS and fixed with 10% neutral buffered formalin for 10 min. The formalin was replaced with PBS, and well fluorescence was read on the VICTOR X3 microplate reader using a 485 nm excitation/535 emission filter set. We developed this methodology, involving CFSE staining of leukocytes and measurement of fluorescence by microplate reader, to ensure objective quantitation that reflected leukocyte binding across the entire endothelial cell monolayer and to facilitate high throughput for large numbers of replicate wells (Figure S2).

### 4.9. Statistical Analysis

Data were analyzed in GraphPad Prism (GraphPad Software, La Jolla, CA, USA). Groups were compared by Student’s *t*-test or one-way or two-way analysis of variance (ANOVA) with Dunnett’s or Tukey’s multiple comparisons test as appropriate to the comparison, implementing pairing when isolates from multiple human donors were involved. In all testing, a statistically significant difference was defined on the basis of a *p*-value less than 0.05.

## Figures and Tables

**Table 1:** Transcripts significantly up-regulated in human retinal endothelial cells following both treatment with IL-1β and treatment with TNF-α for 60 min (n = 5 isolates).

Ensembl Gene ID	Gene Symbol	Description	Treatment with IL-1β		Treatment with TNF-α	
			log2 Fold Change	FDR	log2 Fold Change	FDR
ENSG00000226380	AC016831.1	novel transcript	1.38	1.81 × 10^−7^	1.06	1.83 × 10^−5^
ENSG00000253837	AC090197.1	novel transcript	1.51	2.74 × 10^−7^	1.55	2.51 × 10^−7^
ENSG00000269902	AC234772.2	novel transcript	1.52	2.65 × 10^−4^	1.23	5.35 × 10^−3^
ENSG00000269906	AL606834.1	novel transcript, sense intronic to SAV1	1.19	8.21 × 10^−4^	1.14	1.93 × 10^−3^
ENSG00000279903	AP006248.3	SLC7A2 intronic transcript 1	2.88	2.28 × 10^−5^	2.69	8.16 × 10^−5^
ENSG00000279932	AP006248.4	novel transcript, sense intronic SLC7A2	2.37	3.32 × 10^−4^	2.31	6.35 × 10^−4^
ENSG00000162772	ATF3	activating transcription factor 3	1.87	5.22 × 10^−4^	1.50	7.87 × 10^−3^
ENSG00000095739	BAMBI	BMP and activin membrane bound inhibitor	1.36	2.73 × 10^−3^	1.16	1.89 × 10^−2^
ENSG00000140379	BCL2A1	BCL2 related protein A1	2.49	1.77 × 10^−4^	1.98	5.17 × 10^−3^
ENSG00000023445	BIRC3	baculoviral IAP repeat containing 3	3.13	4.15 × 10^−6^	2.61	6.87 × 10^−5^
ENSG00000159388	BTG2	BTG anti-proliferation factor 2	1.60	2.07 × 10^−5^	1.18	1.74 × 10^−3^
ENSG00000205502	C2CD4B	C2 calcium dependent domain containing 4B	3.56	7.38 × 10^−9^	3.34	3.14 × 10^−8^
ENSG00000108691	CCL2	C-C motif chemokine ligand 2	1.95	1.87 × 10^−3^	1.82	5.51 × 10^−3^
ENSG00000115009	CCL20	C-C motif chemokine ligand 20	6.15	7.28 × 10^−9^	5.14	2.06 × 10^−7^
ENSG00000110848	CD69	CD69 molecule	4.10	1.83 × 10^−9^	3.69	1.40 × 10^−8^
ENSG00000112149	CD83	CD83 molecule	3.29	8.88 × 10^−8^	2.69	2.95 × 10^−6^
ENSG00000179862	CITED4	Cbp/p300 interacting transactivator with Glu/Asp rich carboxy-terminal domain 4	1.47	1.72 × 10^−2^	1.52	2.04 × 10^−2^
ENSG00000164400	CSF2	colony stimulating factor 2	7.54	2.74 × 10^−7^	6.32	7.27 × 10^−6^
ENSG00000006210	CX3CL1	C-X3-C motif chemokine ligand 1	1.18	2.68 × 10^−2^	1.24	2.80 × 10^−2^
ENSG00000163739	CXCL1	C-X-C motif chemokine ligand 1	3.08	3.27 × 10^−6^	2.78	1.89 × 10^−5^
ENSG00000081041	CXCL2	C-X-C motif chemokine ligand 2	5.97	2.73 × 10^−9^	5.07	3.05 × 10^−8^
ENSG00000163734	CXCL3	C-X-C motif chemokine ligand 3	6.15	2.12 × 10^−11^	5.36	2.52 × 10^−10^
ENSG00000169429	CXCL8	C-X-C motif chemokine ligand 8	3.11	1.50 × 10^−5^	2.87	5.41 × 10^−5^
ENSG00000169242	EFNA1	ephrin A1	2.44	8.23 × 10^−7^	2.34	2.64 × 10^−6^
ENSG00000235947	EGOT	eosinophil granule ontogeny transcript	3.34	2.46 × 10^−7^	2.73	9.42 × 10^−6^
ENSG00000179388	EGR3	early growth response 3	1.60	7.94 × 10^−3^	1.40	3.28 × 10^−2^
ENSG00000117525	F3	coagulation factor III, tissue factor	2.39	4.24 × 10^−3^	2.07	2.28 × 10^−2^
ENSG00000125740	FOSB	FosB proto-oncogene, AP-1 transcription factor subunit	2.09	3.44 × 10^−3^	1.78	2.25 × 10^−2^
ENSG00000164949	GEM	GTP binding protein overexpressed in skeletal muscle	2.80	3.87 × 10^−7^	1.98	1.05 × 10^−4^
ENSG00000090339	ICAM1	intercellular adhesion molecule 1	1.52	1.57 × 10^−3^	1.31	9.16 × 10^−3^
ENSG00000137331	IER3	immediate early response 3	2.17	3.52 × 10^−5^	1.68	1.26 × 10^−3^
ENSG00000162783	IER5	immediate early response 5	1.25	1.31 × 10^−3^	1.09	7.95 × 10^−3^
ENSG00000115604	IL18R1	interleukin 18 receptor 1	2.22	4.07 × 10^−5^	1.95	3.22 × 10^−4^
ENSG00000115008	IL1A	interleukin 1 alpha	3.11	4.29 × 10^−6^	2.70	4.97 × 10^−5^
ENSG00000136244	IL6	interleukin 6	2.79	8.46 × 10^−5^	2.29	1.30 × 10^−3^
ENSG00000125347	IRF1	interferon regulatory factor 1	3.21	3.32 × 10^−8^	2.96	2.06 × 10^−7^
ENSG00000171223	JUNB	JunB proto-oncogene, AP-1 transcription factor subunit	1.37	3.66 × 10^−3^	1.21	1.87 × 10^−2^
ENSG00000132510	KDM6B	lysine demethylase 6B	1.93	3.54 × 10^−6^	1.65	5.32 × 10^−5^
ENSG00000237892	KLF7-IT1	KLF7 intronic transcript 1	1.57	2.34 × 10^−7^	1.62	2.14 × 10^−7^
ENSG00000128342	LIF	LIF interleukin 6 family cytokine	3.19	1.78 × 10^−7^	2.26	4.42 × 10^−5^
ENSG00000261618	LINC02605	long intergenic non-protein coding RNA 2605	4.54	1.42 × 10^−8^	3.42	4.85 × 10^−6^
ENSG00000107968	MAP3K8	mitogen-activated protein kinase kinase kinase 8	1.31	5.19 × 10^−3^	1.26	1.01 × 10^−2^
ENSG00000234883	MIR155HG	MIR155 host gene	2.59	9.02 × 10^−6^	2.32	7.05 × 10^−5^
ENSG00000253522	MIR3142HG	MIR3142 host gene	3.00	2.42 × 10^−8^	2.66	2.51 × 10^−7^
ENSG00000163121	NEURL3	neuralized E3 ubiquitin protein ligase 3	1.92	2.65 × 10^−3^	2.16	7.87 × 10^−4^
ENSG00000100906	NFKBIA	NFKB inhibitor alpha	3.06	1.83 × 10^−9^	2.63	1.93 × 10^−8^
ENSG00000144802	NFKBIZ	NFKB inhibitor zeta	2.77	7.63 × 10^−10^	1.78	4.53 × 10^−7^
ENSG00000151014	NOCT	nocturnin	1.91	3.54 × 10^−6^	1.73	2.20 × 10^−5^
ENSG00000163545	NUAK2	NUAK family kinase 2	3.63	2.97 × 10^−9^	3.48	1.40 × 10^−8^
ENSG00000141682	PMAIP1	phorbol-12-myristate-13-acetate-induced protein 1	1.11	8.77 × 10^−4^	1.03	2.82 × 10^−3^
ENSG00000087074	PPP1R15A	protein phosphatase 1 regulatory subunit 15A	1.26	7.28 × 10^−9^	1.04	2.51 × 10^−7^
ENSG00000073756	PTGS2	prostaglandin-endoperoxide synthase 2	1.49	1.81 × 10^−4^	1.23	2.54 × 10^−3^
ENSG00000159200	RCAN1	regulator of calcineurin 1	1.54	4.15 × 10^−6^	1.24	1.23 × 10^−4^
ENSG00000104312	RIPK2	receptor interacting serine/threonine kinase 2	1.33	1.43 × 10^−6^	1.22	9.05 × 10^−6^
ENSG00000172602	RND1	Rho family GTPase 1	2.90	3.68 × 10^−9^	2.78	1.40 × 10^−8^
ENSG00000007908	SELE	selectin E	3.31	1.92 × 10^−3^	3.21	3.67 × 10^−3^
ENSG00000145365	TIFA	TRAF interacting protein with forkhead associated domain	1.84	8.22 × 10^−7^	1.82	1.51 × 10^−6^
ENSG00000133069	TMCC2	transmembrane and coiled-coil domain family 2	1.91	1.75 × 10^−4^	2.00	1.21 × 10^−4^
ENSG00000232810	TNF	tumor necrosis factor	5.92	3.92 × 10^−5^	4.79	1.16 × 10^−3^
ENSG00000185215	TNFAIP2	TNF alpha induced protein 2	1.92	2.00 × 10^−4^	1.36	1.29 × 10^−2^
ENSG00000118503	TNFAIP3	TNF alpha induced protein 3	4.91	4.96 × 10^−9^	4.41	3.14 × 10^−8^
ENSG00000050730	TNIP3	TNFAIP3 interacting protein 3	1.85	1.37 × 10^−3^	1.53	1.72 × 10^−2^
ENSG00000056558	TRAF1	TNF receptor associated factor 1	2.48	7.32 × 10^−4^	2.07	6.07 × 10^−3^
ENSG00000173334	TRIB1	tribbles pseudokinase 1	1.22	5.86 × 10^−3^	1.05	3.28 × 10^−2^
ENSG00000163874	ZC3H12A	zinc finger CCCH-type containing 12A	2.33	8.22 × 10^−7^	1.94	1.89 × 10^−5^
ENSG00000128016	ZFP36	ZFP36 ring finger protein	2.73	2.02 × 10^−6^	2.51	9.42 × 10^−6^

**Table 2:** GeneIDs [55] and sequences for small interfering RNA (Thermo Fisher Scientific-Ambion).

Target Transcript	Gene ID	Sequence (5′-3′)
C2CD4B	388125	Sense: AGAAAGGAAGUACACCCAUTT Antisense: AUGGGUGUACUUCCUUUCUCT
EGR3	1960	Sense: CCAUCAAGGCAUUCAAAGATT Antisense: UCUUUGAAUGCCUUGAUGGTC
FOSB	2354	Sense: ACUUCUUCGUUUGUCCUCATT Antisense: UGAGGACAAACGAAGAAGUGT
IRF1	3659	Sense: CCUCUGAAGCUACAACAGATT Antisense: UCUGUUGUAGCUUCAGAGGTG
JUNB	3726	Sense: CUCUCUACACGACUACAAATT Antisense: UUUGUAGUCGUGUAGAGAGAG

Abbreviations: C2CD4B: C2 calcium dependent domain containing 4B; EGR3: Early growth response 3; FOSB: FosB proto-oncogene, AP-1 transcription factor subunit; IRF1: Interferon regulatory factor 1; JUNB: JunB proto-oncogene, AP-1 transcription factor subunit.

**Table 3:** Primer pairs and product sizes for gene transcripts.

Gene Transcript [Reference]	Primer Pair	Product Size (bp)
ALAS1 [57]	Forward 5′- GGCAGCACAGATGAATCAGA-3′ Reverse 5′- CCTCCATCGGTTTTCACACT-3′	150
C2CD4B [58]	Forward 5′- GCTTGCAACCAGATCCAGAG-3′ Reverse 5′- GGCCGAGGAACAGAGTTTC-3′	113
EGR3 [59]	Forward 5′- GACATCGGTCTGACCAACGAG-3′ Reverse 5′- GGCGAACTTTCCCAAGTAGGT-3′	105
FOSB [60]	Forward 5′- TTCTGACTGTCCCTGCCAAT-3′ Reverse 5′- CGGGGTCAGATGCAAAATAC-3′	249
ICAM-1 [61]	Forward 5′- TAAGCCAAGAGGAAGGAGCA-3′ Reverse 5′- CATATCATCAAGGGTTGGGG-3′	289
IRF1 [62]	Forward 5′- AAAGTCGAAGTCCAGCCGAG-3′ Reverse 5′- CAGAGTGGAGCTGCTGAGTC-3′	103
JUNB [63]	Forward 5′- ACTCATACACAGCTACGGGATACG-3′ Reverse 5′- GGCTCGGTTTCAGGAGTTTG-3′	78
PPIA [64]	Forward 5′- GAGCACTGGAGAGAAAGGATTT-3′ Reverse 5′- GGTGATCTTCTTGCTGGTCTT-3′	355
RPLP0 [64]	Forward 5′- GCAGCATCTACAACCCTGAA-3′ Reverse 5′- GCAGATGGATCAGCCAAGAA-3′	235

Abbreviations: ALAS1: 5′-aminolevulinate synthase 1; C2CD4B: C2 calcium dependent domain containing 4B; EGR3: early growth response 3; FOSB: FosB proto-oncogene, AP-1 transcription factor subunit; ICAM-1: intercellular adhesion molecule 1; IRF1: interferon regulatory factor 1; JUNB: JunB proto-oncogene, AP-1 transcription factor subunit; PPIA: peptidylprolyl isomerase A; RPLP0: ribosomal protein lateral stalk subunit 0.

## Data Availability

All data supporting the reported results are included in the article.

## Supplementary Materials

The supporting information can be downloaded at: https://www.mdpi.com/article/10.3390/ijms24043304/s1.
