# Supplementary material for: Selective Transcription Factor Blockade Reduces Human Retinal Endothelial Cell Expression of Intercellular Adhesion Molecule-1 and Leukocyte Binding
Source: Int J Mol Sci. 2023 Feb 7;24(4):3304. doi: 10.3390/ijms24043304 (PMC9967456; doi:10.3390/ijms24043304)
Supplement: Supplementary file 1 [file ijms-24-03304-s001.zip › Supplementary_Figure_S2.pdf]

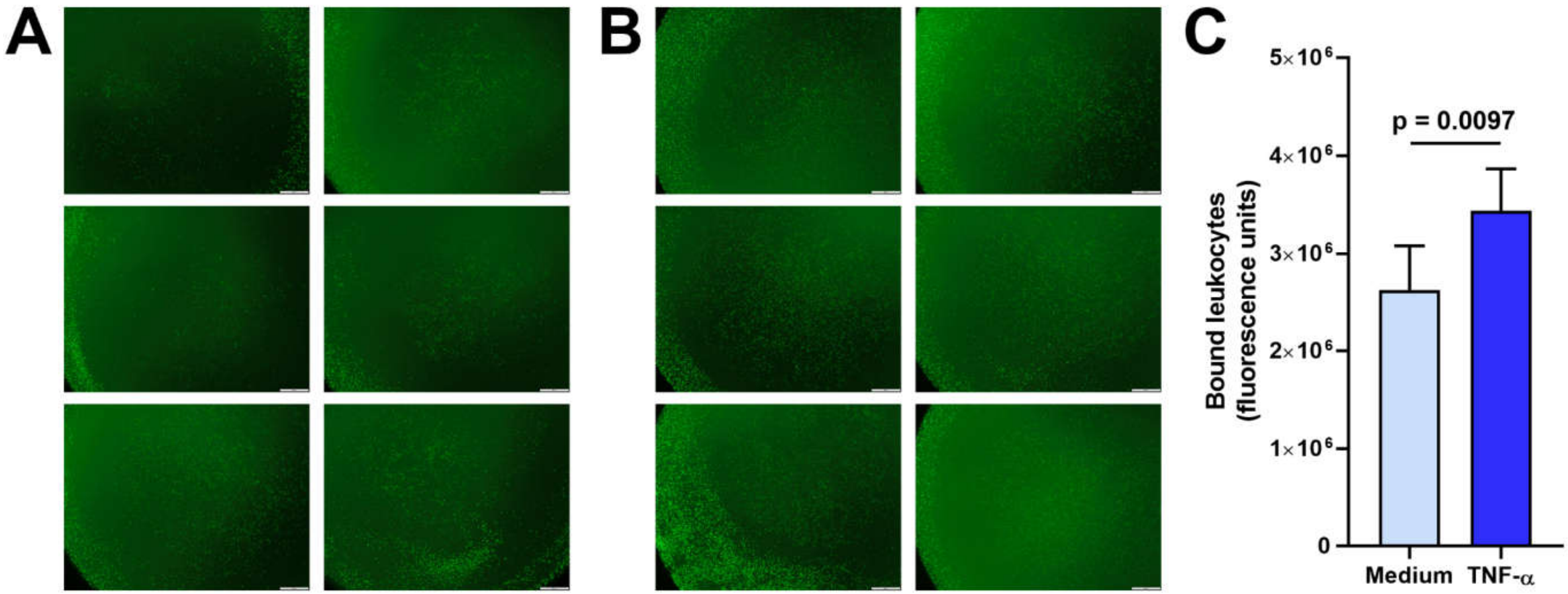

Figure S2. Leukocyte binding assay. (A & B) Epifluorescence photographs showing wells of a 96-well multi-well plate taken at the end of the assay, with CFSE-labelled leukocytes bound to non-labelled retinal endothelial cell monolayers that had been exposed for 24 hours to (A) fresh medium alone or (B) TNF-α. White bars indicate 200 µm. (C) Graph showing microplate reader quantitation of leukocyte binding to endothelial cell monolayers in the same wells, expressed in fluorescence units. Bars indicate mean, and error bars indicate standard deviation ($n = 6$ endothelial cell monolayers per condition). Data were analyzed by unpaired Student's t-test.
