# Supplementary material for: Selective Transcription Factor Blockade Reduces Human Retinal Endothelial Cell Expression of Intercellular Adhesion Molecule-1 and Leukocyte Binding
Source: Int J Mol Sci. 2023 Feb 7;24(4):3304. doi: 10.3390/ijms24043304 (PMC9967456; doi:10.3390/ijms24043304)
Supplement: Supplementary file 1 [file ijms-24-03304-s001.zip › Supplementary_Figure_S1.pdf]

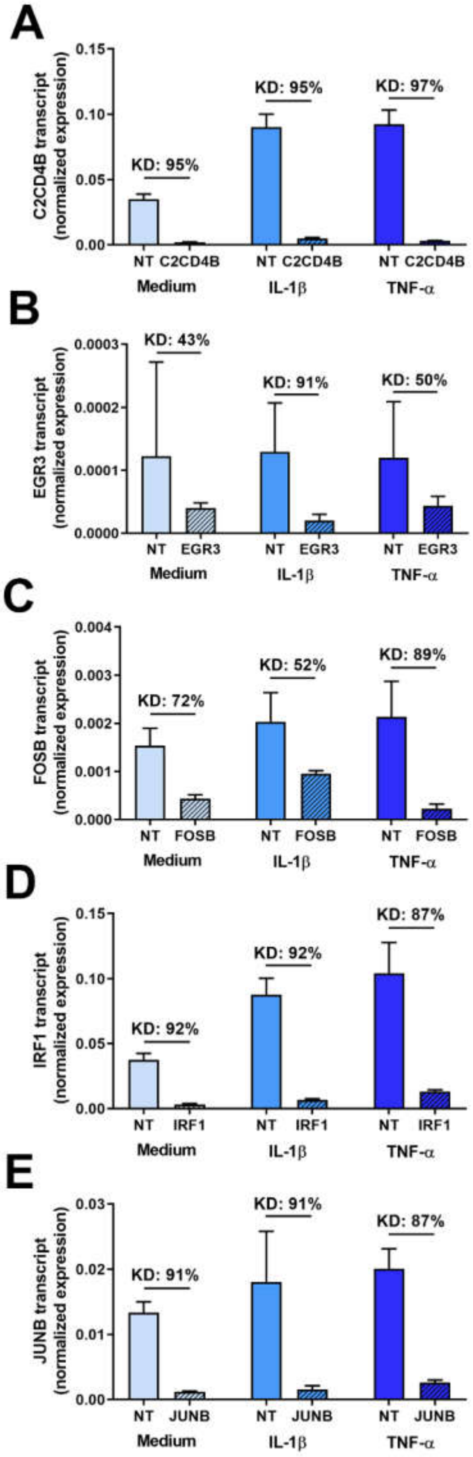

Figure S1. RNA silencing. Graphs showing normalized expression of (A) C2CD4B, (B) EGR3, (C) FOSB, (D) IRF1 and (E) JUNB transcript, calculated relative to ALAS and PPIA, in human retinal endothelial cells treated for 48 hours with transcription factor-targeted or control non-targeted (NT) siRNA, and subsequently treated with IL-1β or TNF-α, or fresh medium alone for 24 hours. Bars indicate mean, and error bars indicate standard deviation (n = 3-4 endothelial cell monolayers per condition). KD represents percentage knockdown.
